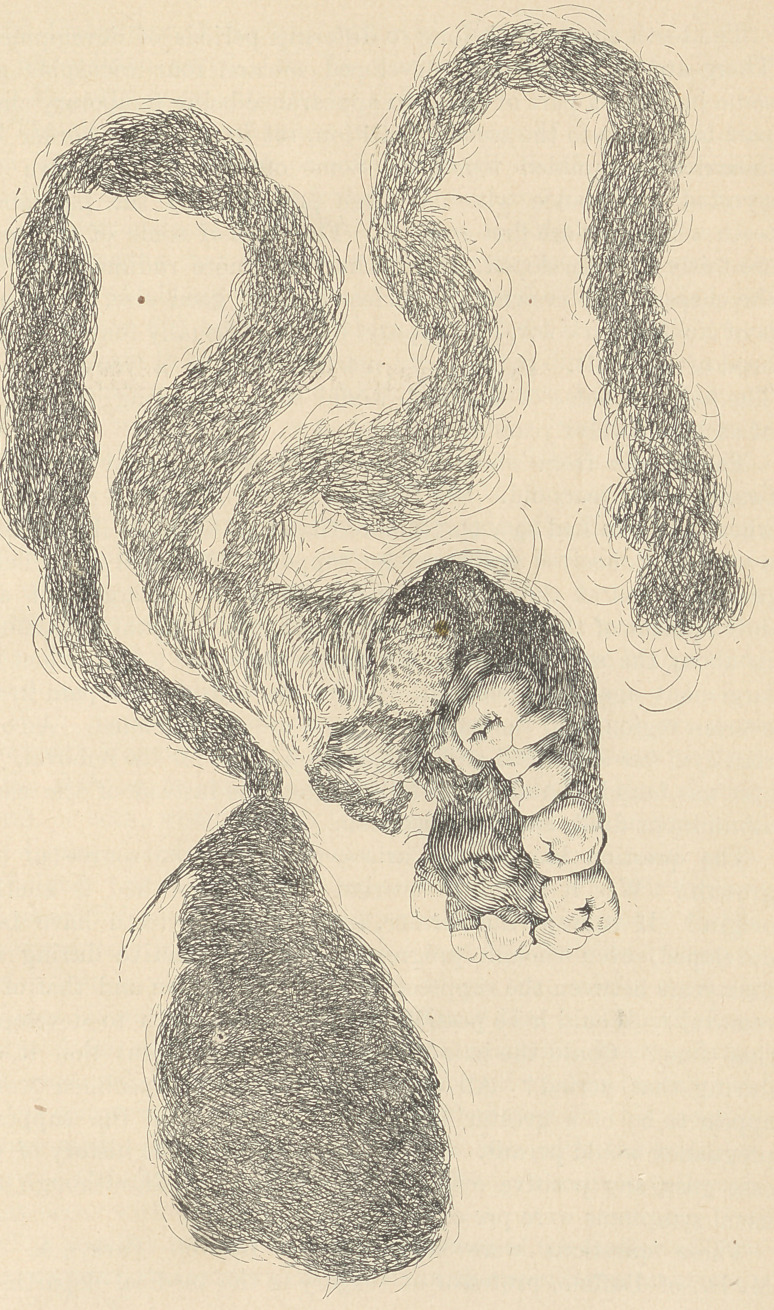# An Ovarian Cyst

**Published:** 1887-06

**Authors:** W. C. Barrett


### AN OVARIAN CYST

BY DR. W. C. BARRETT.

The remarkable specimen, of which an illustration is given opposite, was exhibited to a few dentists at the last meeting of the American Dental Association, at Niagara, by Dr. C. B. Hewitt, of Kansas City. It was placed in my hands for illustration, with the expectation that the surgeon who removed it would prepare a full statement of its history to accompany the cut. This he has failed to do, and the cut is therefore given with as much of the history as I have been able to procure.

The specimen consists of a considerable amount of hair, some of which is nearly a foot in length, what resembles a partially developed superior maxillary bone, and twelve teeth. The posterior portion of the mass from which the hair depends bears some resemblance to an occipital bone. The mass is represented in the cut a little less than its actual size.

The teeth appear to belong to differing periods of development. There are three very well developed molars, four bicuspids and some incisors, a part of them in appearance being temporary and a part belonging to the second dentition, set in no regular order but inextricably jumbled together. Some of the incisors have the serrated appearance which is often presented by the permanent teeth when they are first erupted. The roots of some of the teeth are fairly well developed, while others are more rudimentary, but the cusps of the crowns are perfect and of good size. The hair is of an auburn color, and appears like that usually found on the head of an adult. It is clearly not like that of a young infant. The hair and some of the teeth would seem to belong to a person of at least twelve years of age.

The woman from whom this specimen was removed was about twenty-seven years old. She had miscarried in her first pregnancy, when her term had something more than half expired. In less than a year she conceived again, as she supposed, the usual signs being present. But at the end of four months the enlargement and development of the supposed fœtus ceased, and everything remained in about the same condition for three years and a half, when enlargement again commenced. There was considerable pain in the ovarian region, and the patient became much emaciated. An ovarian cyst was diagnosed and an operation made for its removal. A cyst was found, containing a milky substance instead of the usual albuminous fluid, and this specimen.

The question immediately arises, what was the source of this growth? Was it a case of ovarian fœtation, or had it another origin? If it were the former, how could the teeth have been developed, even though progression had been active during the four years between the supposed term of conception and that of its removal? Would it be possible for permanent teeth to develop in that time? Could the hair have grown to the extent that it did during that period? All of the tissues in the specimen would appear to be of a greater age than the history of the supposed pregnancy would permit. Nor is there, so far as the history of the case goes, any positive evidence of pregnancy at all, although the usual symptoms were present for a short time.

These questions were submitted to Roswell Park, A. M., M. D., of Buffalo, professor of surgery in the medical department of the University of Buffalo, editor of *The Medical Press*, etc., and his opinion asked. He has kindly furnished THE INDEPENDENT PRACTITIONER with the following:

"The above described tumor plainly belongs to the class of dermoid cysts, which are notoriously frequent about the ovary and testicle, though they may be found in almost any location, one having been demonstrated in the brain. It is one of the peculiarities of these growths that they most often contain fat, hair, teeth and skin, all of which are products of the external blastodermic layer of the embryo, and even bone, which comes from the middle layer. For many years these tumors were held to be products of a conception which had failed to reach the fallopian tubes, but in view of later researches this view is no longer tenable. Such cysts are always congenital in origin, though they may be late in development. In the case in question, the growth of the tumor was probably excited by the physiological activity accompanying the first pregnancy; it advanced slowly, provoked such symptoms as to lead to a suspicion of a second pregnancy, halted in its course for a number of months, as any tumor may, and then resumed active growth. The milky fluid which it contained, would doubtless have been found, upon analysis, to contain cholesterine in abundance.

"With regard to their more exact method of origin we must agree, with Heschel and His, that they arise from isolated portions of the epiblast or mesoblast (external and middle blastodermic layers), or both, which during the development of the embryo have been displaced and located somewhere where they do not properly belong. Such islands of tissue retain nearly all their embryonal possibilities, and, given an impetus, such as pregnancy in the above case, may in the early or adult life of the individual begin to develop into any or all of the tissues which they might normally produce. It was especially His, who showed that in the part of the embryo from which the internal genital organs are developed, the three germinal layers are exceedingly complicated and combined, and the comparative frequency of dermoid tumors in this location is thus made clearer. This corresponds also with the inclusion theory of Cohnheim. A dermoid cyst is always a monocyst, though it may occur with others.

"The peculiar interest in this case, to most of the readers of THE INDEPENDENT PRACTITIONER, will lie in the peculiar arrangement of the teeth, those resembling deciduous and permanent being promiscuously mixed. We may hold, I think, that a dental germ under abnormal conditions may develop, according to its surroundings, in various abnormal ways. Assuming that a permanent tooth must have, say fifteen years for proper growth, we have here a patient of twenty-seven, with a tumor which *must* have had its beginnings in her own fœtal life. Assuming further, that it made no progress till she had attained the age of puberty, which is by no means assuming too much, but is most probable, we yet have sufficient time for the full growth of a permanent tooth. And this, too, on the assumption that because such a tooth *usually* requires so much time it *inevitably* does, which is by no means proven. Probably the deciduous teeth noticed in the specimen were of later growth; nevertheless, we must not expect obedience to the ordinary laws in such apparently lawless growths, where all development notoriously goes on with seeming inconsistency.

"While in reality we yet know very little about dermoid cysts, we are sufficiently familiar with them to assign the above case and specimen its proper place, which, I take it, is an advance made rather recently."

## Figures and Tables

**Figure f1:**